# A comparative study of intraoral versus retromandibular approach in the management of subcondylar fracture

**DOI:** 10.1186/s12893-019-0487-7

**Published:** 2019-03-05

**Authors:** Seung Min Nam, Yong Bae Kim, Sun Jae Lee, Eun Soo Park, Jang Hyun Lee

**Affiliations:** 1 Department of Plastic and Reconstructive Surgery, College of Medicine, Soonchunhyang University, 170 Jomaru-ro, Bucheon, 14584 Republic of Korea; 2 Department of Plastic and Reconstructive Surgery, Hanyang University, College of Medicine, 153, Gyeongchun-ro, Guri, 11923 Republic of Korea

**Keywords:** Fracture fixation internal, Mandible, Mandibular condyle, Mandibular fractures, Oral surgical procedures

## Abstract

**Background:**

The purpose of this study was to compare the outcomes and effectiveness between intraoral approach and retromandibular approach for treatment of subcondylar fracture of mandible.

**Methods:**

Between March 2011 and October 2013, 24 patients with subcondylar fractures of the mandible were treated by a single surgeon with an intraoral approach using an angulated screwdriver (*n* = 14) or by another surgeon using a retromandibular approach (*n* = 10). The interincisal distance was measured 1 week (T0), 6 weeks (T1), 3 months (T2), and 6 months (T3) postoperatively. We also compare the average operation time and the cost of operation between the two groups.

**Results:**

At 6 months postoperatively, all 24 patients achieved satisfactory ranges of temporomandibular joint movement, with an interincisal distance > 40 mm without deviation and with stable centric occlusion. The intraoral group had the median interincisal distance of 14 mm at T0, 38 mm at T1, 42.5 mm at T2, and 43 mm at T3, while the retromandibular group had that of 15, 29, 35, and 42.5 mm respectively. There was no statistically significant difference between the intraoral and the retromandibular group at T0 and T4. However, significant differences were noted T1 and T2 (*p* < 0.01). The differences of average operation time between the intraoral (81 min) and retromandibular group (45 min) were statistically significant (*p* < 0.01). The cost of an operation was 369.96 ± 8.14 (United States dollar [USD]) in intraoral group and was 345.48 ± 0.0 (USD) in retromandibular group. The differences between the two groups were statistically significant (*p* < 0.01).

**Conclusion:**

In open reduction of a subcondylar fracture of the mandible, a intraoral approach using an angulated screwdriver is superior to the retromandibular approach in terms of interincisal distance, although the operation time is longer.

## Background

Mandibular fractures, including fractures of the subcondylar and condylar regions, are common facial fractures [1]. Subcondylar fractures account for 20–62% of all mandibular fractures [2–4] but their management remains controversial [5–7]. Although closed reduction is the most useful method, it can be difficult to achieve anatomical reduction with this technique compared with open reduction and internal fixation (ORIF) [6–8]. The most surgeons agreed the consensus that the proper surgical indications for ORIF are the displaced bilateral or unilateral fractures of the mandibular condylar neck or subcondyle [9–11].

Among the numerous surgical methods that can be used in the treatment of subcondylar fracture [11–16], extraoral rather than intraoral approaches are generally preferred because they can be provided a sufficient surgical vision. However, compared with intraoral approaches, extraoral approaches commonly have a high rate of postoperative complications, such as salivary fistula formation, visible scarring, and facial nerve injury [6, 7, 16–18].

We previously reported the clinical outcomes of patients with subcondylar fractures of the mandible treated with ORIF through a modified intraoral approach with an angulated screwdriver [19]. However, the effectiveness of that technique in the present setting is unclear. Therefore, in this study we compared the clinical results achieved with our intraoral approach using an angulated screwdriver versus a retromandibular approach in patients with subcondylar fractures of the mandible, who were treated by ORIF.

## Methods

Between March 2011 and October 2013, 24 patients with subcondylar fractures of the mandible were treated either by one surgeon (S.M. Nam) using an intraoral approach with an angulated screwdriver (*n* = 14) or by another surgeon (E.S. Park) using a retromandibular approach (*n* = 10). The inclusion criteria were an age older than 15 years and presentation with a displaced subcondyle and occlusion disturbances. Patients with contralateral condylar or subcondylar fractures or condylar neck fractures were excluded from this study. The subcondylar fractures of mandible are defined the fracture line is positioned below the level of the most inferior part on the sigmoid notch. [7] The study conformed to the principles dictated by the Declaration of Helsinki, and written consent was obtained from each patient for both the surgery and the publication of photographs of the results. The study was reviewed and approved by the Institutional Review Board for Human Subject Research (SCHBC 2017–01–002-002) and informed consent was obtained from each patient.

Before the miniplate osteosynthetic fixation of subcondylar fracture, under local anesthesia, the madibulomaxillary fixation (MMF) was performed to achieve the centric occlusion using skeletal fixation with bone-anchoring skeletal self-drilling screws (Dual-Top Anchor System; Jeil Medical, Seoul, Korea) and the rubber bands in all patients. After the ranged from 3 to 7 days, the rigid and internal fixation of subcondylar fracture wad performed. In preoperative clinical examination, the dental occlusion was evaluated with clinical examination and a review of photograph was taken using self-cheek retractor. The direction and type of fracture fragments were evaluated preoperatively with computed tomography.

Patients were taken a liquid diet until the postoperative 7 days and educated to maintain a soft diet for the next 4 weeks. The MMF was maintained until postoperative 7 days and then, after the rubber bands were removed, mouth-opening physiotherapy was performed. The patients can be coming back to daily life at postoperative 8–9 days. The absorbable sutures in the intraoral approach were stitched out at postoperative 7–9 days and the non-absorbable Nylon sutures in the retromandibular approach were stitched out 5 days postoperatively. The bone-anchoring skeletal self-drilling screws were removed at postoperative 6 weeks in outpatient clinics.

### Surgical procedure

#### The retromandibular approach

The surgical technique was similar to that described by Ellis and Dean [16], Ebenezer et al. [20]. Briefly, a 3–3.5-cm incision was made 0.5 cm below the earlobe, not extending below the angle of the mandible. A blunt metzenbaum scissors was used to achieve blunt dissection through the subcutaneous tissue to the parotid capsule, which was then incised to allow dissection through the parotid gland, parallel to the branches of the facial nerve. When the latter were encountered, they were first carefully dissected and then retracted to minimize tension. The periosteum was incised at the posterior border of the mandibular ramus. After subperiosteal dissection of the ramus and subcondylar areas, reduction and fixation of the fracture segments were achieved. The mandibular ramus was retracted inferiorly using manual pressure, which resulted in sufficient working space for reduction of the fracture segments using one or two 2.0-mm miniplates. Special attention was paid to complete closure of the parotid capsule. After the occlusion was confirmed and the wound was copiously irrigated, the wound was repaired.

#### The intraoral approach with an angulated screwdriver system

Our modified intraoral approach with an angulated screwdriver was described in a previous report [19]. Briefly, an intraoral incision was made in the mucosa overlying the external oblique ridge. The subperiosteal dissection was performed under the masseter muscle from the lateral to the posterior border of the ascending mandibular ramus and the insertion of the temporalis muscle was detached from the coronoid process. Through this procedure, it could be provided a sufficient operative space and surgical vision for the ORIF of subcondylar fracture. Using an Obwegeser channel retractor, it could be visualized from the sigmoid notch to the mandibular angle of the entire surface of the ramus. The fracture site and dislocated condylar segment were also identified.

**Fig. 1:**
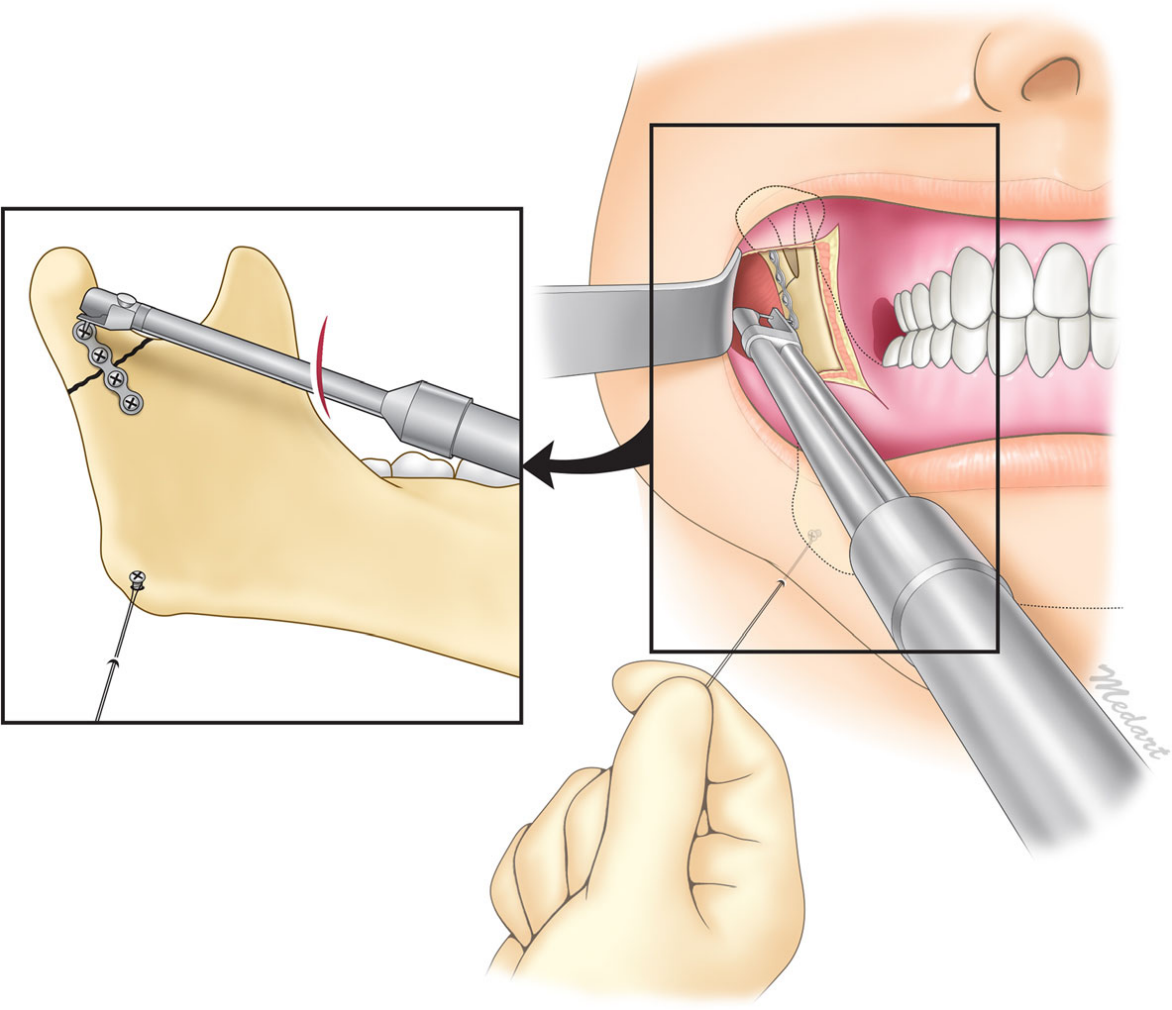
Illustration of the operative procedure for treatment of a subcondylar fracture using intraoral approach with an angulated screwdriver. Reprinted from “Transoral open reduction for subcondylar fractures of the mandible using an angulated screwdriver system” by Nam SM, Kim YB, Cha HG, Wee SY, Choi CY, *Annals of Plastic Surgery*, 2015, 75(3), 295–301 [19]. Copyright by Wolters Kluwer Health

An 11-mm titanium screw was placed at the mandibular angle to hang the wire used the inferior ramus retractor. A stab incision was made using an 18-G needle in the 1 cm inferior to the mandible to allow insertion of a 27-gauge wire. The wire was hung on the titanium screw to be used the inferior retractor of the ramus. During the manual inferior traction of ramus, the displaced subcondylar fracture segment was reduced using a periosteal elevator. When the fracture segment was well positioned within the mandibular fossa, we slowly released the manual inferior traction of the ramus to maintain reduction status (Fig. 1).

After fracture reduction was confirmed using an angulated mirror, MMF was applied to maintain the centric occlusion. The fracture fragment can be maintained through the Obwegeser channel retractor was positioned under the inferolateral border of the condyle during fixation of the plate and screw. The four-hole 2.0-mm titanium miniplate was placed at the perpendicular position to the fracture site and was fixed with the 6 mm screw at the proximal fracture segment using an angulated screwdriver. Then, with the plate pulled to eliminate the bone gap of fracture site, it was fixed with the screw onto the mandibular ramus and then additionally fixed with screw in remained 2 holes. After the wound was irrigated and bleeding control conducted, we confirmed the centric occlusion and the stability of internal fixation at the fracture site.

### Clinical examination

**Fig. 2:**
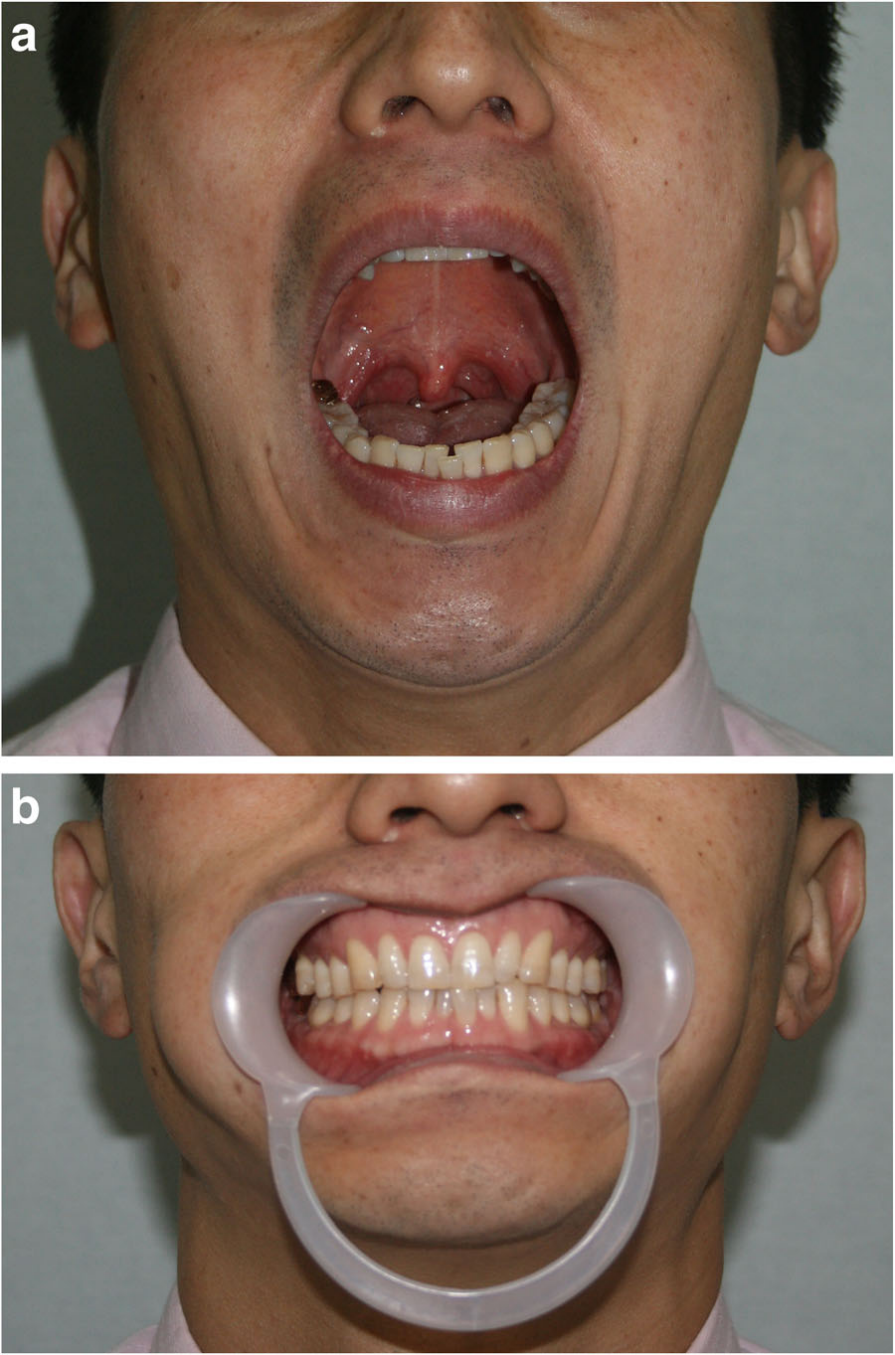
The interincisal distances were evaluated after surgery. In this patient, the interincisal distance was 38 mm 6 weeks after surgery (**a**) and the patient had neutron-occlusion postoperatively (**b**)

The interincisal distance was evaluated at 1 week(T0), 6 weeks(T1), 3 months(T2), and 6 months(T3) after surgery and the occlusion was evaluated (Fig. 2). The average operation times of the intraoral and retromandibular groups were determined from the respective anesthesia records. The cost of an operation analysis between intraoral and retromandibular groups was performed with direct payment data.

Clinical and radiological evaluations were performed during the postoperative follow-up period. Clinical outcomes of both approaches were evaluated with the occlusion status, range of mouth opening (and deviation), wound infection, nonunion, and plate and screw loosening or exposure.

### Statistical analysis

Statistical analyses were performed using SPSS version 20.0 (SPSS Inc., Chicago, IL, USA). The Mann-Whitney U test was used to compare the interincisal distances, the average operation time and the operation cost of each approach. A *p* value < 0.05 was considered to indicate statistical significance.

## Results

Fourteen patients with subcondylar fractures of the mandible were treated via a intraoral approach using an angulated screwdriver. In this group, three patients had isolated subcondylar fractures of the mandible and 9 patients had other mandibular fractures. Among the 10 patients with subcondylar fractures treated via a retromandibular approach, 7 had isolated subcondylar fractures and 3 patients had other mandibular fractures (Table 1). During the follow-up period (range, 6–10 months), no major postoperative complication was encountered. There was no surgical site infection, bleeding, plate exposure or fracture, bony absorption or condylar necrosis, or salivary fistula. One patient in the retromandibular group had immediate postoperative facial expression weakness but full recovery occurred within 3 months postoperatively.

**Table 1:** Demographic characteristics of patients

Variable	Intraoral approach	Retromandibular approach	*p-*value^a^
Sample size (*n* = 24)	14 (58.3)	10 (41.7)	
Sex (male)	13 (92.9)	7 (70.0)	0.139
Associated mandibular fracture			
None	3 (21.4)	7 (70.0)	
Symphysis	6 (42.9)	1 (10.0)	
Parasymphysis	5 (35.7)	2 (20.0)	0.062
Injury side (right)	5 (35.7)	5 (50.0)	0.484
Age (years)	36.71 ± 4.16	29.30 ± 3.34	0.151

Data are presented as number (%) or mean ± standard deviation

^a^*p*-values were computed by Chi-square test or Fisher’s exact test for categorical variables and Mann-Whitney U test for continuous variables

**Fig. 3:**
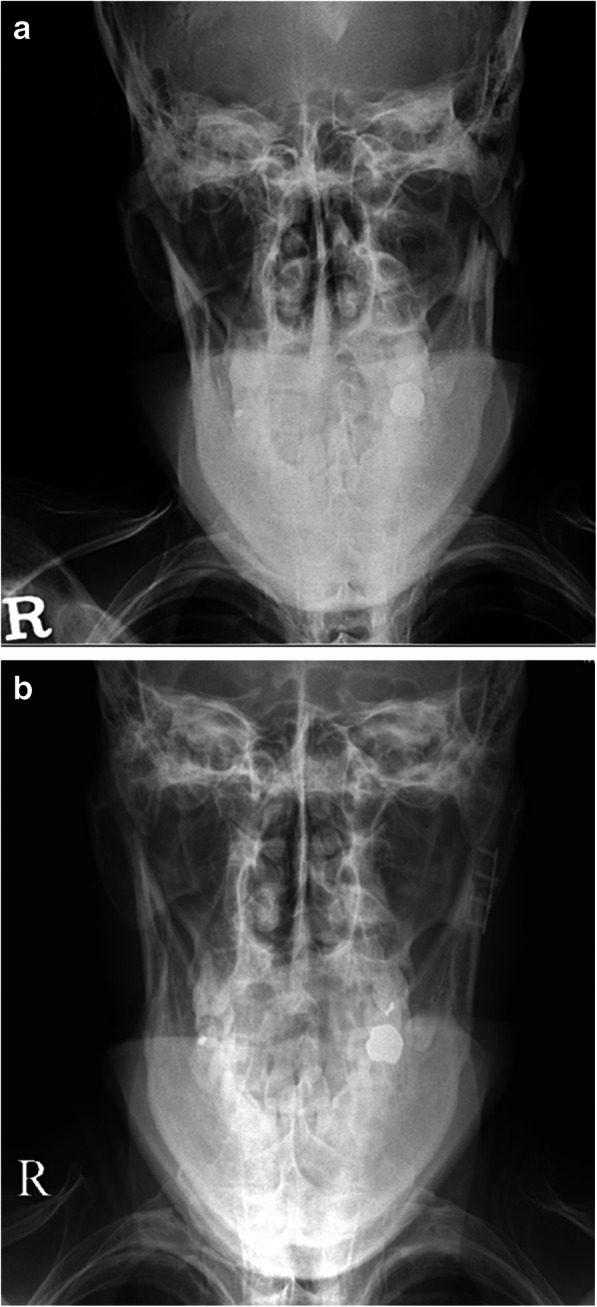
A 40 year-old male with a left subcondylar fracture of the mandible was treated using the retromandibular approach. Modified Towne’s radiographic images show the medially deviated subcondylar fracture (**a**) and the postoperative reduction state of the fracture following rigid internal fixation (**b**)

**Fig. 4:**
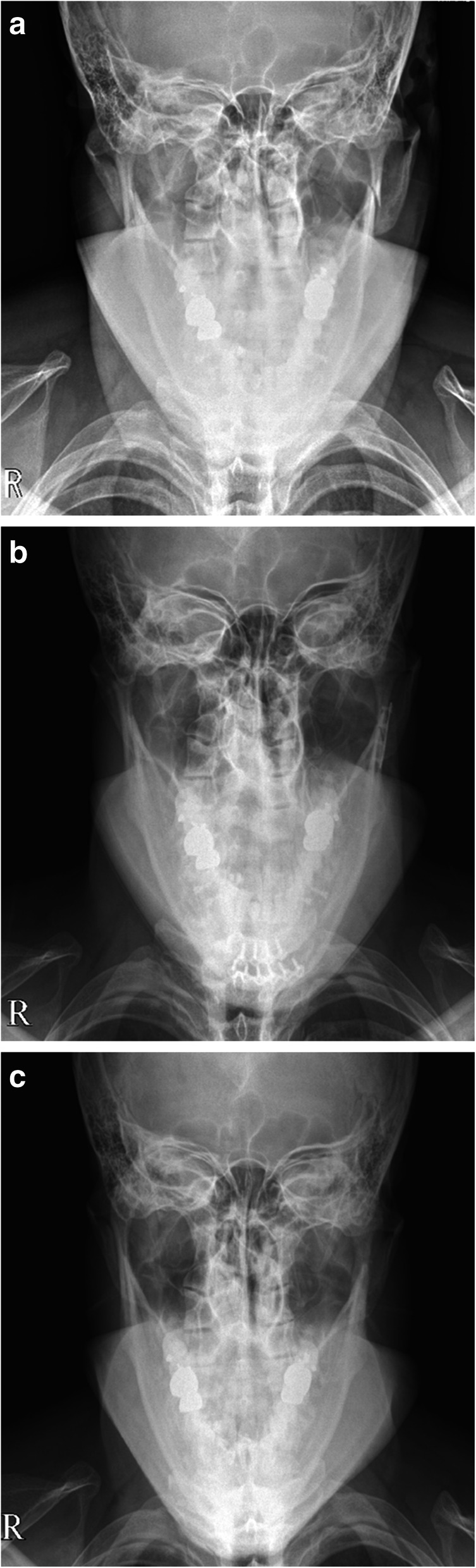
A 31 year-old male with a left subcondylar fracture of the mandible was treated using the modified intraoral approach. Modified Towne’s radiographic images show a laterally deviated subcondylar fracture (**a**) and the postoperative reduction state of the fracture following rigid internal fixation (**b**). Eight months after surgery, the plate and screws were removed through an incision made at the previous oral incision scar, without an external incision (**c**)

Radiological follow-up was performed using computed tomography, Towne’s and mandibular radiographic series, and orthopantomography, which reduction and fixation of all fractures in both groups (Figs. 3 and 4). Towne’s and mandibular radiographic series were performed at 1 week, 6 weeks, 3 months and 6 months after surgery and the computed tomography was performed at 3 months after surgery. The orthopantomography was performed at 1 day postoperatively. Clinical examination revealed ipsilateral deviation on mouth opening in one patient in the transoral group and one in the retromandibular group for about 1 month postoperatively. These patients were treated with mouth-opening physiotherapy at an outpatient clinic and gradually improved. At 6 months postoperatively, all patients had satisfactory ranges of TMJ movement, interincisal distances of > 40 mm without deviation, and stable centric occlusion. There were no clinically problematic symptoms related to TMJ movement in all patients.

**Fig. 5:**
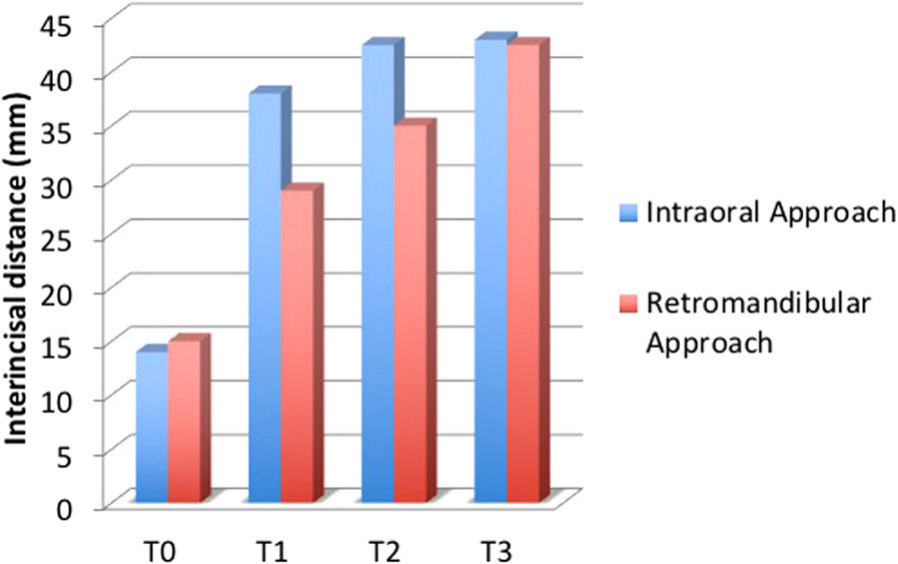
Interincisal distances achieved using the intraoral and retromandibular approaches. The differences 6 weeks (T1) and 3 months (T2) after the operation were statistically significant (*p* < 0.01)

At 1 week postoperatively, the median interincisal distances were 14 mm (13–15 mm) in the intraoral group and 15 mm (15–16 mm) in the retromandibular group. At postoperative 6 weeks, the median interincisal distances were 38 mm (37–39 mm) and 29 mm (29–30 mm), respectively. After 3 months, the median interincisal distances were 42.5 mm (41–44 mm) in the intraoral group and 35 mm (34–35 mm) in the retromandibular group. After 6 months, the corresponding values were 43 mm (42–45 mm) and 42.5 mm (41–44 mm). The differences between the two groups were statistically significant at 6 weeks and 3 months (*p* < 0.01) but not at 1 week or 6 months postoperatively (Fig. 5 and Table 2).

**Table 2:** Comparison of interincisal distance according to surgical approach

	Interincisal distance (mm)		*p-*value^a^
	Intraoral approach	Retromandibular approach	
1 week (T0)	14 (13–15)	15 (15–16)	0.07
6 weeks (T1)	38 (37–39)	29 (29–30)	< 0.01
3 months (T2)	42.5 (41–44)	35 (34–35)	< 0.01
6 months (T3)	43 (42–45)	42.5 (41–44)	0.403

^a^*p*-value calculated using Mann-Whitney U test

**Fig. 6:**
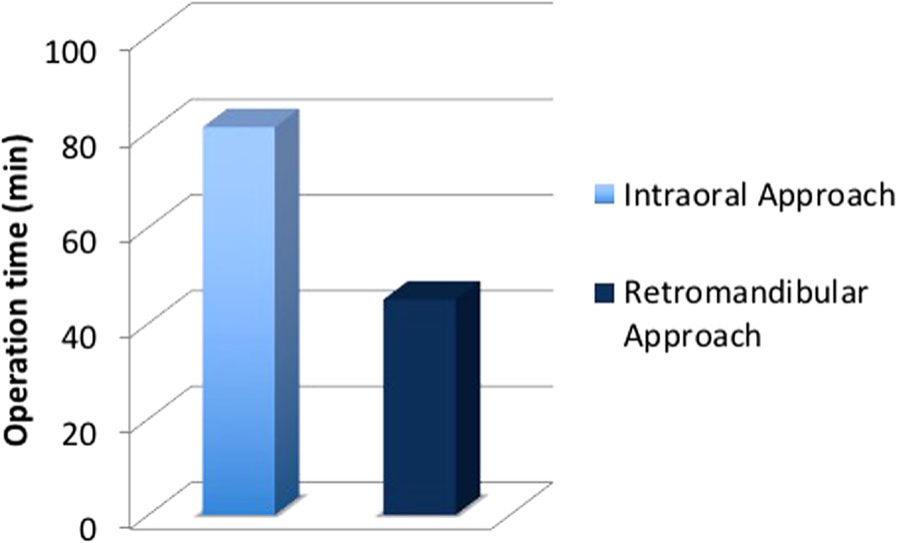
Average operation times of the intraoral and retromandibular approaches. The difference between the two procedures was significant (*p* < 0.01)

Based on the anesthesia records, the average operation time in the retromandibular group was significantly shorter than in the intraoral group (*p* < 0.01). The average operation time in retromandibular group was 45 min and in intraoral group was 81 min (Fig. 6 and Table 3).

**Table 3:** Comparison of operation time according to surgical approach

	Intraoral approach	Retromandibular approach	*p-*value^a^
Operation time (min)	81 (65–100)	45 (40–45)	< 0.01

^a^*p*-value calculated using Mann-Whitney U test

**Fig. 7:**
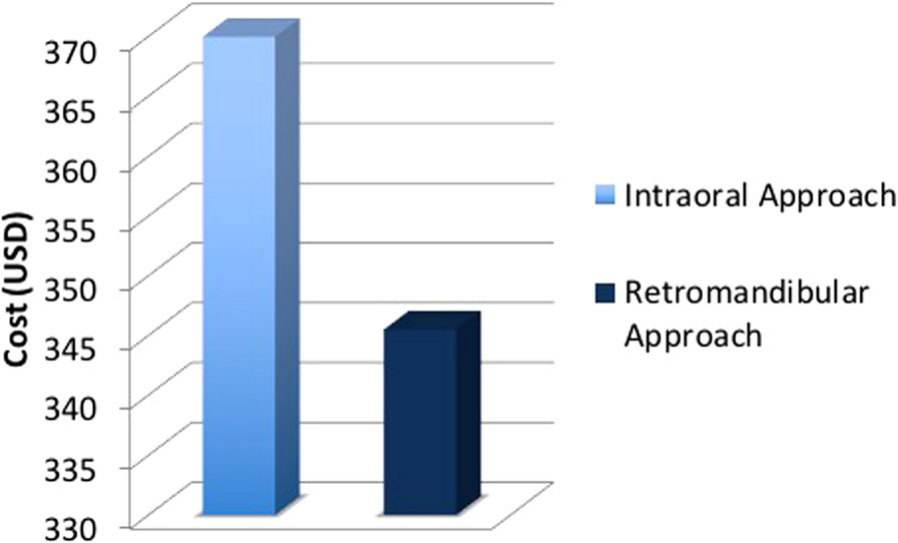
Comparison of operation cost between the intraoral and retromandibular approaches. The difference between the two procedures was significant (*p* < 0.01). *USD* United States dollar

The cost of an operation in intraoral group was 369.96 ± 8.14 (USD) and in retromandibular group was 345.48 ± 0.0 (USD). The differences between the two groups were statistically significant (*p* < 0.01) (Fig. 7 and Table 4).

**Table 4:** Comparison of operation cost according to surgical approach

	Intraoral approach	Retromandibular approach	*p*-value^a^
Operation cost (USD)	369.96 ± 8.14	345.48 ± 0.0	< 0.01

Data are presented as mean ± standard deviation

^a^*p*-value calculated using Mann-Whitney U test

*USD* United States dollar

## Discussion

Nondisplaced subcondylar fractures of the mandible are usually treated with a closed procedure and MMF. In such cases, short-term MMF stimulates the callus formation, which prevents mobility of fracture segments. After the MMF is removed, mouth-opening physiotherapy is initiated [21, 22]. The advantages of closed treatment were the uneventful healing and a minimal risk of long-term disability or pain [21–24]. However, it can lead to the open-bite deformity or malocclusion in displaced subcondylar fractures due to the shortening of the ascending ramus [25–28]. In such cases, open reduction is therefore recommended [10, 16, 28–31]. Nonetheless, the indications for surgical treatment of displaced fractures are still debatable due to the postoperative scar and the possibility of facial nerve injury during external approaches [6, 16, 32–34]. However, surgical treatment of lower neck and subcondylar fractures may be better than closed treatment with respect to postoperative mouth opening [35].

In this study, only patients with subcondylar fractures of the mandible were included. Patients with fractures above the condylar neck were excluded because, in these fractures, a intraoral approach does not provide sufficient space to allow fixation of the plate. Moreover, surgical treatment of fractures above the condylar neck can result in limited TMJ movement and prevent the correct evaluation of interincisal distances.

The retromandibular approach has several advantages. First, it provides an excellent operative field and direct visual alignment of the fracture fragments [36, 37], with exposure of the entire fracture from the posterior border to the condylar process. Second, it allows for a reduction in the distance from the skin incision to the fracture site and for fixation of the plate without the need for a transcutaneous trocar [38]. Third, it avoids damage to the facial nerve [39]. Although facial expression weakness sometimes occurs in patients treated via a retromandibular approach, this may be caused by facial muscle weakness rather than nerve damage [36]. Immediate weakness of the facial nerve has been described in some retromandibular treated patients, but full recovery occurred within 3 months [34, 40]. In other studies, no similarly treated patient had facial nerve weakness [39–41]. Salivary fistula is a potential complication of the retromandibular approach [34, 42, 43]. In our study, watertight closure of the parotid capsule prevented the formation of salivary fistulas.

The surgical indications for an intraoral approach are considered to be limited to moderately dislocated subcondylar or mandibular ramus fractures, because, otherwise, there is insufficient surgical space to allow the use of an angulated screwdriver [35]. However, the inferior traction of the ramus using can be provided the sufficient surgical to allow performance of a modified intraoral approach with an angulated screwdriver, as shown in this and our previous study [19]. In addition to the adequate surgical space, an important advantage of our modified intraoral approach using an angulated screwdriver is that it eliminates the risk of external scarring, including scarring inflicted during removal of the palate and screw, and avoids both facial nerve damage and salivary fistula formation.

A comparison of interincisal distances achieved using the intraoral and retromandibular approaches showed significant differences only at 6 weeks and 3 months (*p* < 0.01), thus not at 1 week or 6 months, postoperatively. The significant differences in interincisal distances between the time points may be attributable to soft-tissue scarring. Although the dissection range of the intraoral approach is wider than that of the retromandibular approach, no skin and soft-tissue scarring develops in patients treated intraorally, whereas, in the retromandibular approach, scarring occurs in the skin, soft tissue, and parotid fascia.

The difference in operation time between the intraoral and retromandibular approaches was statistically significant (*p* < 0.01), which may reflect the greater technical difficulty of the former procedure. However, after advanced training, surgeons may be able to perform ORIF through an intraoral approach alone [16, 44].

The difference in operation cost between the intraoral and retromandibular approaches was statistically significant (*p* < 0.01). Although it was statistically significant difference, the difference of operation cost between intraoral and retromandibular approaches was only 25 dollars under Korean national health care system. Because the payment of subcondylar fracture is already decided regardless of the approach of subcondylar fracture and the payment of anesthesia is charged in one-hour intervals in Korean national health care system. When the patients who want to avoid facial scar choose surgical approach, this difference will not be a consideration. The limitations of our study were that the retromandibular and intraoral procedures were performed by two surgeons rather than by a single surgeon. In addition, only a small number of patients, with relatively restricted surgical indications (fractures of the condylar neck and subcondylar fractures of the mandible) were included.

Despite these limitations, we were able to show that our modified intraoral approach with an angulated screwdriver is superior to a retromandibular approach in treatment of subcondylar fracture. First, the patients were treated by the modified intraoral approach could be felt the convenience of daily life from 6 weeks to 3 months after surgery, because it could be provided wider interincisal distances compared to retromandibular approach. Second, modified intraoral approach remained very small stab incision scar that commonly cannot be identified. This is an important advantage in East Asian population that wants to avoid visible scar.

## Conclusion

This study suggests that, in the treatment of subcondylar fractures of the mandible using open reduction, an intraoral approach using an angulated screwdriver is superior to a retromandibular approach, based on the interincisal distance achieved. However, at least initially, the intraoral approach involves a longer operation than the retromandibular approach.

## Abbreviations

MMF: mandibulomaxillary fixation
ORIF: open reduction and internal fixation
TMJ: temporomandibular joint
